# Comparison of laparoscopic-assisted radical vaginal hysterectomy and abdominal radical hysterectomy in patients with early stage cervical cancer: A retrospective study

**DOI:** 10.1097/MD.0000000000008005

**Published:** 2017-09-08

**Authors:** Sichen Zhang, Linlin Ma, Qing Wei Meng, Dan Zhou, Tuerhongayi Moyiding

**Affiliations:** Department of Gynecology and Obstetrics, Beijing Hospital, Dong Dan, Beijing, P.R. China.

**Keywords:** abdominal radical hysterectomy, early stage cervical cancer, laparoscopic-assisted radical vaginal hysterectomy, survival outcomes

## Abstract

**Background:**

The aim of this study was to compare the safety and survival outcomes of early stage cervical cancer patients treated by laparoscopically assisted radical vaginal hysterectomy (LARVH) versus abdominal radical hysterectomy (ARH).

**Methods:**

Since March 2008 to July 2012, the patients with early stage cervical cancer undergoing LARVH or ARH in Beijing hospital have been entered into this study. Statistical analysis used Statistical Product and Service Solutions (SPSS) and significance was defined as *P* < .05.

**Result:**

Forty-two patients were included in LARVH group and 35 patients in ARH group. Both groups were similar with respect to age, body mass index (BMI), histological diagnosis, and stage. There were no differences in operative time, vaginal length, and postoperative complications, but blood loss, rate of transfusion, length of catheterized, and length of hospital stay were significantly less in LARVH. Number of lymph node retrieved was less than ARH. No differences were seen regarding recurrence rate, length of disease free survival, overall survival, and mortality rate after a median follow up of 58.5 and 48.5 months.

**Conclusion:**

LARVH is a suitable alternative to ARH for early-stage cervical cancer, which shows less blood loss, shorter catheterized and hospital stay, and similar survival outcomes.

## 1 Introduction

Abdominal radical hysterectomy^[1,2]^ has been the mainstay of surgery for early-stage cervical cancer since Wertheim^[3,4]^ reported in 1900 and Schauta^[5]^ developed the vaginal radical hysterectomy at the same time. Dargent^[6]^ first described the combination of Schauta operation with endoscopy in 1987. The technical feasibility of laparoscopically assisted radical vaginal hysterectomy (LARVH) as treatment for early-stage cervical cancer has been well established through a series of institution,^[7–16]^ which suggest that LARVH may have an intraoperative reduction in blood loss, transfusion requirement, and hospital stay but longer operative time. But just a few authors^[8,12,13,15]^ reported the survival outcomes which was most important question to evaluate the prognosis of therapy.

The purpose of this study was to compare the feasibility, safety, and survival outcomes of early stage cervical cancer women treated by LARVH to women treated by abdominal radical hysterectomy (ARH).

## 2 Material and methods

Between March 2008 and July 2012, 77 women underwent LARVH or ARH for International Federation of Gynecology and Obstetrics (FIGO2009) stage IA2 to IIB cervical cancer in Beijing Hospital. Women were informed of the experimental nature of the operation and offered open radical hysterectomy as an alternative. The operation type was type III radical hysterectomy including resection of uterus and parametrium 3 cm and vagina 3 cm, besides pelvic lymph node dissection. Those who consented for counselling underwent LARVH, others underwent ARH. The reason why for LARVH or ARH chosen was, first, patients and families required the LARVH or ARH on the premise of surgeons informing. Second, patients who suffered in HBsAg(+) (Hepatitis B surface antigen), HIV(+) (human immunodeficiency virus), HCV(+) (Hepatitis C virus), or Tp-Ab(+) (Syphilis antibody) could not underwent LARVH on account of laparoscopy being not accepted. There were 2 gynecological oncology surgeons who complete all surgery together.

Data were collected retrospectively from the charts as follows: age, body mass index (BMI), histological diagnosis, stage, operative time, blood loss, transfusion rate, number of lymph node dissection, resection of vaginal length, catheter retain, postoperative complications, hospital stay, and recurrence data including length of follow up, recurrence rate, length of disease free survival, overall survival rate, and mortality rate.

Results were analysis using SPSS (Statistical Product and Service Solutions, SPSS Inc., IL) version 10.0 software. BMI, operative time, blood loss, number of lymph node dissection, resection of vaginal length, catheter retain, and hospital stay were compared with the 2-independent *t* test. Age and length of follow up were compared with Wilcoxon rank-sum test. Histological diagnosis, stage, transfusion, postoperative complications were compared with χ^2^ test. Recurrence-free survival data were calculated from the date of surgery, and distributions used the product-limit method of Kaplan and Meier. Differences between survival curves were compared with the log rank test. Statistical significance was defined as *P* < .05.

Ethical approval statement: all the data from the patients had been informed. The date ethical committee approval was June 18th and registration number was 2015BJYYEC-037-01.

## 3 Results

Between March 2008 and July 2012, 42 and 35 patients have undergone ARH by 4 surgeons and LARVH by 1 surgeon respectively for FIGO2009 IA2 to IIB cervical cancer. As shown in Table 1, patients and controls were comparable in age, BMI, histological diagnosis, and stage and none of them had significant difference.

**Table 1:**
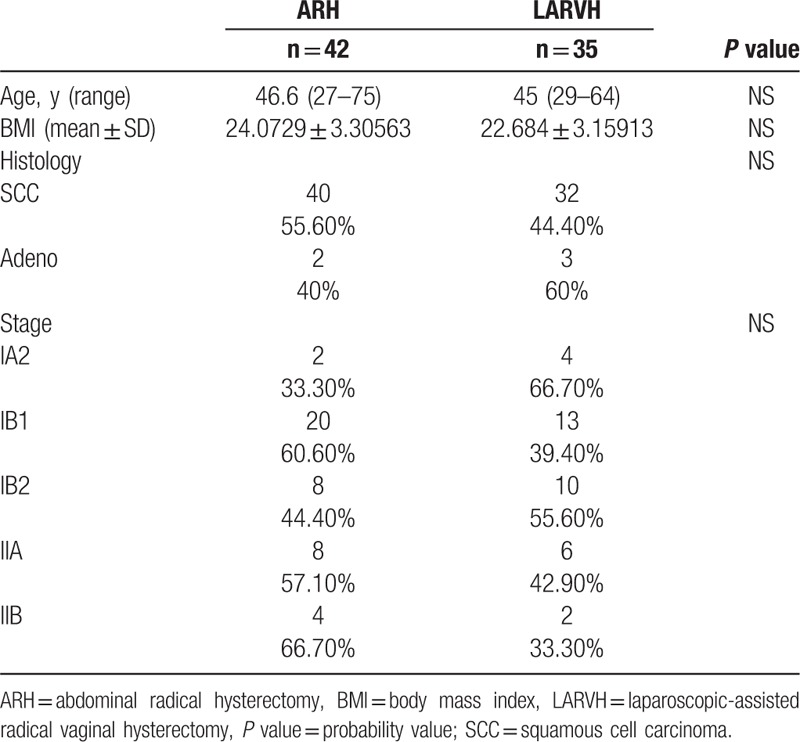
Patient characteristics.

Table 2 describes the intraoperative factors. The blood loss was greater in the ARH (861.91 ± 349.36 vs 502.86 ± 236.69, *P* < .05) reaching statistical significance, and so did the transfusion rate (54.8% vs 8.6%), showing a significant trend as well. The number of lymph nodes retrieved was more in the ARH (36.19 ± 12.28 vs 23.71 ± 9.45, *P* < .05). The vaginal length was similar for both groups and matched the standard required by the current guidelines. Postoperative complications are listed in Table 3. Postoperative complications included 6 cases of lymphocyst in ARH group versus 5 in LARVH, no case of retention of urine in ARH versus 2 in LARVH, and 2 cases of venous thromboembolism. However, the ARH patients were catheterized for a mean of 10.57 ± 4.59 days compared with 7.83 ± 2.56 days in the LARVH group (*P* < .05). The mean postoperative hospital stay was significantly shorter for the LARVH than that for the ARH patients (21.41 ± 14.94 vs 14.37 ± 11.78 days, *P* < .05).

**Table 2:**
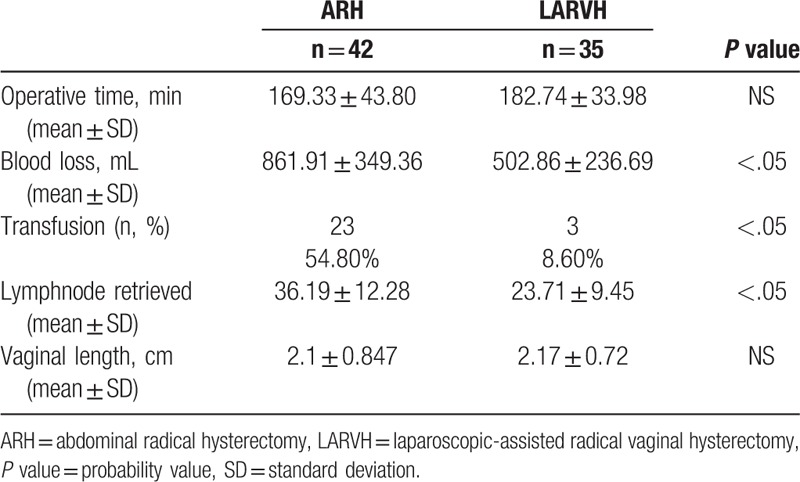
Intraoperative factors.

**Table 3:**
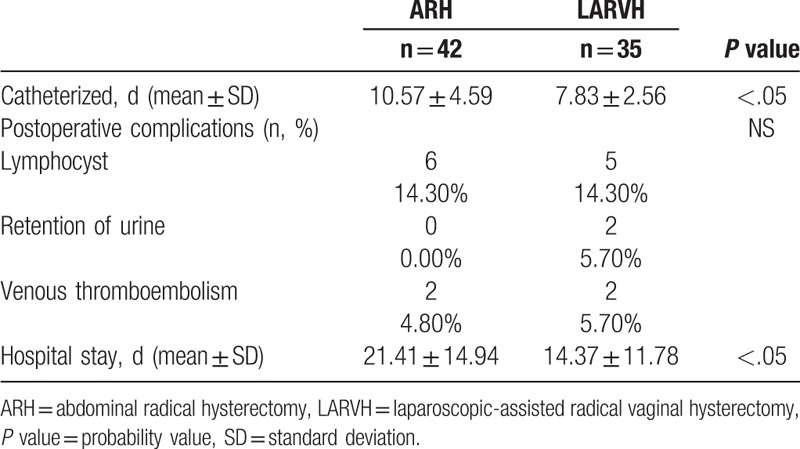
Postoperative factors.

The cases which more than stage IB1 or suffered more than 2 high risk factors (tumor size >4 cm, lymph node positive, margin positive, parametrium positive, and vessel carcinoma) were treated by radiation therapy after operation. There were 24 patients in ARH while 20 patients in LARVH underwent radiation therapy after operation. Twenty patients were more than stage IB1 and 3 patients and 1 patient occupied 2 and 3 high risk factors in ARH group. Meanwhile, 18 patients were more than stage IB1 and 2 patients possessed 2 high risk factors in LARVH.

**Figure 1:**
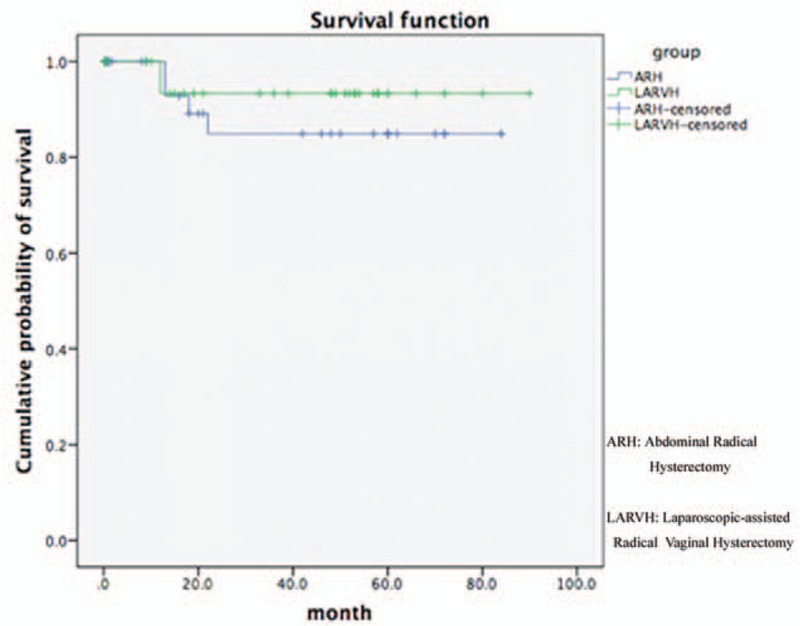
Survival function of ARH and LARVH. ARH = abdominal radical hysterectomy, LARVH = laparoscopically assisted radical vaginal hysterectomy.

After a median follow-up of 48.5 (range, 19–84 months) months for LARVH and 58.5 (range, 16–84 months) months for ARH, 3 patients lost in LARVH, and 12 patients lost in ARH. Ten ARH patients (33.33%) and 3 LARVH patients (9.4%) developed recurrent disease, which showed the recurrence rate in ARH was significantly higher than LARVH (*P* < .05). The mean of length of disease free survival (DFS), overall survival rate, and mortality rate showed no difference between LARVH and ARH, respectively (Table 4) (Fig. 1).

**Table 4:**
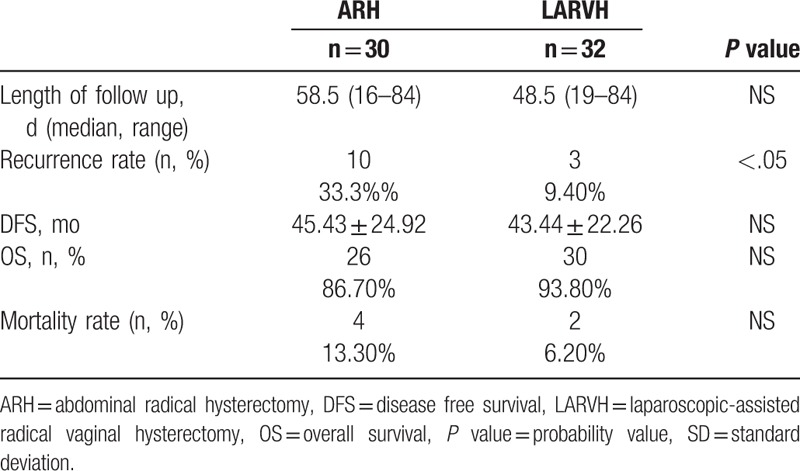
Recurrences and survival outcomes.

## 4 Discussion

The current procedure performed for patients with early stage cervical cancer are ARH, laparoscopic radical hysterectomy (LRH), laparoscopically assisted radical vaginal hysterectomy (LARVH), and robotic radical hysterectomy (RRH).^[17,18]^ Many studies have shown that LARVH is associated with less intra- and postoperative complications universally.^[7–11,13–16]^ However, in China no one reported the long-term prognosis of LARVH maybe because of new technique without long time follow up. In our study, we reported the survival outcomes based on long-term follow up which was scarce data.

Our results supported the proposed benefits of less blood loss, transfusion rate, shorter catheterized, and hospital stay. The estimated blood loss was less than in LARVH reaching statistical differences, as transfusion requirement showed significantly less than ARH. Length of hospital stay was significantly shorter in LARVH group. It could be taken as a reflection of a shorter and less painful recovery. The same goes for length of catheterized, which compared favorably in LARVH, and differences between LARVH and ARH were statistically significant.

In previously reported by others, mean operating time was significantly greater in LARVH versus ARH patients. The other researchers pointed out reason of “early phase” of learning curve and time was shorter than the initial patients after increased experience. Moreover, this fact reflexed the main drawbacks of LARVH—difficult learning process for surgeons in sufficiently skilled on vaginal surgery. The vaginal process required some tactile and purely intuitive skills on tissue management and dissection which was different from visual teaching. The most technical challenge was the identification of vaginal cuff (especially in case after cervical conization) and ureters inside the bladder pillars vaginally. However, in our study, the difference of mean operating time between LARVH and ARH considered no statistically significant. The surgeon in Beijing Hospital controlled the skill after few cases which depended on experiences of numerous vaginal route surgery such as vaginal hysterectomy. On the other side to explain the operating time was less number of lymph node retrieved in LARVH. Lymphadenectomy was new technique for the surgeon and the lymph tissue contained multiple small blood vessels which need meticulously coagulated. In initial patients, surgeon retrieved <15 lymph node, but number of lymph node was similar with ARH after experience increased. However, the vaginal length was similar between LARVH and ARH which demonstrated the technical vaginal surgery skill for surgeon secondly.

Some investigator reported higher rate postoperative complications in LARVH compared with ARH which was not the case in our case. The most frequent was lymphocyst which perhaps is related to technical skill in lymph node retrieved. Other complications reported in the literature included retention of urine and venous thromboembolism. While there was no statistical difference between LARVH and ARH for each postoperative complication.

Only a few authors in the current literature have reported outcomes and recurrence rates after LARVH. The median of length of follow up in each literature was no more than 40 months. In our result, the median length of follow up was 58.5 months (range, 16–84 months) in ARH and 48.5 months (range, 19–84 months) in LARVH which had no significant difference, except lost 12 patients in ARH and 3 patients in ARH. However, the recurrence rate, length of disease free survival, overall survival rate, and mortality rate were similar to each other. It is correctly that only a prospective randomized trial could produce a scientific evidence testing equivalence which require a prohibitive sample size of approximately 14,000 patients.

The limitation of this study was the sample size and single center study. All the early stage cervical cancer cases (77 cases) underwent radical hysterectomy from 2009 (time for LARVH beginning) to 2012 (time for follow-up beginning) were included. We need more cases and other centers around the world, properly controlled prospective trials are required to resolve the issues of postoperative and long-term prognosis such as this study, to more clearly define the true place of LARVH in cervical cancer surgery.

In conclusion, our study supported the use of the LARVH for early stage cervical cancer patients. The procedure had distinct advantages of minimally invasive access surgery and similar survival outcomes in comparison to ARH.
